# Perioperative chemotherapy with docetaxel plus oxaliplatin and S-1 (DOS) versus oxaliplatin plus S-1 (SOX) for the treatment of locally advanced gastric or gastro-esophageal junction adenocarcinoma (MATCH): an open-label, randomized, phase 2 clinical trial

**DOI:** 10.1007/s10120-024-01471-z

**Published:** 2024-03-08

**Authors:** Zhichao Jiang, Yibin Xie, Wen Zhang, Chunxia Du, Yuxin Zhong, Yuelu Zhu, Liming Jiang, Lizhou Dou, Kang Shao, Yongkun Sun, Qi Xue, Yantao Tian, Shugeng Gao, Dongbing Zhao, Aiping Zhou

**Affiliations:** 1 Department of Medical Oncology, National Cancer Center/ National Clinical Research Center for Cancer/ Cancer Hospital, Chinese Academy of Medical Sciences, and Peking Union Medical College, No. 17, Panjiayuannanli Street, Chaoyang District, Beijing, 100021 China; 2 Department of Pancreatic and Gastric Surgery, National Cancer Center/ National Clinical Research Center for Cancer/ Cancer Hospital, Chinese Academy of Medical Sciences and Peking Union Medical College, Beijing, 100021 China; 3 Department of Pathology, National Cancer Center/ National Clinical Research Center for Cancer/ Cancer Hospital, Chinese Academy of Medical Sciences and Peking Union Medical College, Beijing, 100021 China; 4 Department of Imaging Diagnosis, National Cancer Center/ National Clinical Research Center for Cancer/ Cancer Hospital, Chinese Academy of Medical Sciences and Peking Union Medical College, Beijing, 100021 China; 5 Department of Endoscopy, National Cancer Center/ National Clinical Research Center for Cancer/ Cancer Hospital, Chinese Academy of Medical Sciences and Peking Union Medical College, Beijing, 100021 China; 6 Department of Thoracic Surgery, National Cancer Center/ National Clinical Research Center for Cancer/ Cancer Hospital, Chinese Academy of Medical Sciences and Peking Union Medical College, Beijing, 100021 China

**Keywords:** Gastric cancer, Perioperative chemotherapy, DOS, SOX

## Abstract

**Background:**

It remains unclear whether addition of docetaxel to the combination of a platinum and fluoropyrimidine could provide more clinical benefits than doublet chemotherapies in the perioperative treatment for locally advanced gastric/gastro-esophageal junction (LAG/GEJ) cancer in Asia. In this randomized, phase 2 study, we assessed the efficacy and safety of perioperative docetaxel plus oxaliplatin and S-1 (DOS) versus oxaliplatin plus S-1 (SOX) in LAG/GEJ adenocarcinoma patients.

**Methods:**

Patients with cT3–4 N_any_ M0 G/GEJ adenocarcinoma were randomized (1:1) to receive 4 cycles of preoperative DOS or SOX followed by D2 gastrectomy and another 4 cycles of postoperative chemotherapy. The primary endpoint was major pathological response (MPR).

**Results:**

From Aug, 2015 to Dec, 2019,154 patients were enrolled and 147 patients included in final analysis, with a median age of 60 (26–73) years. DOS resulted in significantly higher MPR (25.4 vs. 11.8%, *P* = 0.04). R0 resection rate, the 3-year PFS and 3-year OS rates were 78.9 vs. 61.8% (*P* = 0.02), 52.3 vs. 35% (HR 0.667, 95% CI: 0.432–1.029, Log rank *P* = 0.07) and 57.5 vs. 49.2% (HR 0.685, 95% CI: 0.429–1.095, Log rank *P* = 0.11) in the DOS and SOX groups, respectively. Patients who acquired MPR experienced significantly better survival. DOS had similar tolerance to SOX.

**Conclusions:**

Perioperative DOS improved MPR significantly and tended to produce longer PFS compared to SOX in LAG/GEJ cancer in Asia, and might be considered as a preferred option for perioperative chemotherapy and worth further investigation.

**Supplementary Information:**

The online version contains supplementary material available at 10.1007/s10120-024-01471-z.

## Introduction

Gastric cancer (GC) is the fifth most commonly diagnosed cancer globally and the fourth leading cause of cancer death [1]. In China, it holds the third highest incidence and mortality rates [2]. Moreover, a substantial proportion of Chinese GC patients, approximately 70.8%, are diagnosed with locally advanced disease [3].

The early MAGIC and FNCLCC/FFCD studies established the perioperative chemotherapy modality in local advanced gastric or gastro-esophageal junction (LAG/GEJ) cancer. These studies first proved that pre- and post-operative chemotherapy with epirubicin, cisplatin, and fluorouracil (ECF) and cisplatin and fluorouracil (FP) significantly improved the disease-free survival (DFS) and overall survival (OS) compared with surgery alone [4, 5]. The JCOG0501 study failed to confirm that the perioperative chemotherapy with SP is superior to adjuvant S1 alone [6]. However, two Asian randomized clinical trials demonstrated the advantage of perioperative chemotherapy over postoperative chemotherapy alone. Perioperative oxaliplatin plus S-1 (SOX) and docetaxel combined with oxaliplatin and S-1 (DOS) have been shown to significantly improve the 5-year OS rate compared to adjuvant chemotherapy in RESOLVE and PRODIGY studies, respectively [7, 8]. The perioperative chemotherapy strategy has gained increased evidence and well accepted by most Asian countries.

Although, currently, immune checkpoint inhibitors (ICIs) and targeted therapies are being extensively investigated in the perioperative treatment and have shown promising results in phase II/III clinical trials, chemotherapy remains the cornerstone of neoadjuvant treatment in gastric cancer, and there is still a need to optimize the regimen in terms of efficacy, safety, and convenience. The docetaxel, oxaliplatin, and 5-FU (FLOT) regimen was proved significantly superior to the old ECF in terms of major pathological response (MPR) and survival, and has become the standard perioperative regimen in Europe, as supported by the FLOT4 study [9]. Due to the preference of Asian countries for using oral fluoropyrimidine drugs such as capecitabine or S-1, platinum-based doublet chemotherapies, such as SOX, CapOX, and SP, are the most commonly used regimens in metastatic gastric cancer as well as in the neoadjuvant setting [6, 7, 10, 11], while FLOT has not been widely used in Asia. Among them, the SOX regimen, supported by the RESOLVE study, is considered the preferred perioperative chemotherapy for LAG/GEJ cancer in China [12]. Whether the FLOT regimen and its triplet regimen analogues, consisting of a taxane combined with a platinum and oral fluoropyrimidine drugs, such as DOS, DOX, or DCS, could offer additional clinical benefits compared to their doublet counterparts of SOX, CapOX, or CS, has not been thoroughly investigated yet. In a phase II study with a small sample (*N* = 74), FLOT did not show a significant advantage in MPR compared to the SOX regimen (20 vs. 34%, *P* = 0.289), and the survival outcomes of both arms were not reported [13].

As early as 2015, we initiated the MATCH study, a two-component, randomized phase 2 clinical trial (NCT02725424). The aim was to evaluate the efficacy and safety of the DOS and SOX regimens in HER2-negative patients with LAG/GEJ cancer. Additionally, we assessed the therapeutic effects and safety of the combination of the SOX with trastuzumab compared to the SOX alone in HER2-positive patients. Here we present the outcomes of the HER2-negative component of the MATCH study.

## Methods

### Study design and objective

This was a single-center, open-label, randomized phase II study which aimed to evaluate the efficacy and safety of DOS versus SOX as perioperative chemotherapy in Chinese patients with LAG/GEJ adenocarcinoma conducted at National Cancer Center, China. The protocol was approved by the Ethical Committee of the National Cancer Center/ National Clinical Research Center for Cancer/ Cancer Hospital, Chinese Academy of Medical Sciences, and Peking Union Medical College (15-077/1004). Clinical trial information: NCT02725424 (https://classic.clinicaltrials.gov/ct2/show/NCT02725424).

### Patients

The main inclusion criteria were: (1) age ≥18 years; (2) patients with histologically diagnosed G/GEJ (Siewert II/III type) adenocarcinoma; (3) HER2-negative; (4) cT3–4 Nany M0 by CT (AJCC 7th Edition); (5) no previous treatment including chemotherapy, radiotherapy or gastrectomy; (6) Eastern Cooperative Oncology Group (ECOG) performance status of ≤1; (7) adequate hematological, hepatic and renal functions.

The exclusion criteria were: (1) a diagnosis of other malignant tumors (except for cured skin cancer or cervical carcinoma in situ) within five years; (2) allergic to any ingredients of docetaxel, oxaliplatin, or S-1 medications; (3) distant metastatic disease.

### Procedures

Patients were randomly assigned (1:1) to receive perioperative DOS or SOX via an automated interactive web response system. Randomization was stratified by the primary tumor’s location (gastric vs. GEJ cancer), lymph node status (N0 vs. N+), and Lauren classification (intestinal vs. diffuse vs. mixed).

Four cycles of pre- and post-operative chemotherapy with the DOS and SOX regimens were administered. In the DOS group, during each 21-day treatment cycle, patients received intravenous docetaxel 60 mg/m^2^ and oxaliplatin 100 mg/m^2^ on day 1, oral S-1 twice a day depending on body surface area (BSA) (BSA < 1.25 m^2^, 80 mg/day; BSA ≥ 1.25 to <1.5 m^2^, 100 mg/day and BSA ≥ 1.5 m^2^, 120 mg/day) from day 1 to day 14. In the SOX group, oxaliplatin was given intravenously at a dose of 130 mg/m^2^ on day 1, and the S-1 dosing schedule was the same as that of the DOS group, repeated every 21 days. Radical gastrectomy with D2 lymphadenectomy was performed within 4–6 weeks after preoperative chemotherapy.

Preoperative tumor assessment was performed by CT of the chest, abdomen, and pelvis every two cycles according to RECIST version 1.1. Baseline and pre-surgery evaluations such as MRI of the stomach, gastroscopy, and endoscopic ultrasound were recommended but not mandatory. The pathological response was evaluated according to the Becker TRG system by a specified independent pathologist, with tumor regression classified as follows: Grade 1a: complete regression; Grade 1b: <10% residual tumor per tumor bed; Grade 2: 10–50% residual tumor per tumor bed; Grade 3: >50% residual tumor per tumor bed. Adverse events were assessed by the National Cancer Institute Common Terminology Criteria for Adverse Events (version 4) both prior to and after the surgery.

### Outcomes

The primary endpoint was an MPR analyzed in the modified intention to treat (mITT) population. MPR was defined as pathological complete (TRG1a) and subtotal regression (TRG1b) of the primary tumor according to the Becker TRG system. Secondary endpoints included the 3-year progression free survival (PFS) rate, 3-year OS rate, R0 resection rate, pCR (ypT0N0M0), and safety. PFS was defined as the time from randomization to the first occurrence of disease progression or recurrence based on radiological diagnosis, or death from any cause, whichever occurs first. OS was defined as the time from randomization to death. R0 resection was defined as complete tumor resection without macroscopic or microscopic residual disease.

### Statistical analysis

All analyses were performed using SPSS software ver. 25. The MPR in the SOX group was estimated to be 15%, and a calculated sample size of 70 patients per group was needed assuming an improvement in MPR by DOS of 35%, with *α*level of 0.05 (two-sided) and test power of 0.8. Considering a drop-out rate of 5%, 74 patients per group were required. Patients who withdrew their consent before undergoing preoperative chemotherapy were excluded from the analysis. The remaining patients, who were randomized and received any form of study treatment, were included in the mITT population. The MPR, survival, chemotherapy safety, and resection analyses were conducted in the mITT population, while the pathological stage and postoperative complication analyses were performed on patients who underwent surgery (surgery population). PFS and OS were analyzed by the Kaplan–Meier method. Categorical data between these two groups were compared using the chi-squared test. All P-values were 2-sided.

## Results

### Patient characteristics

**Fig. 1:**
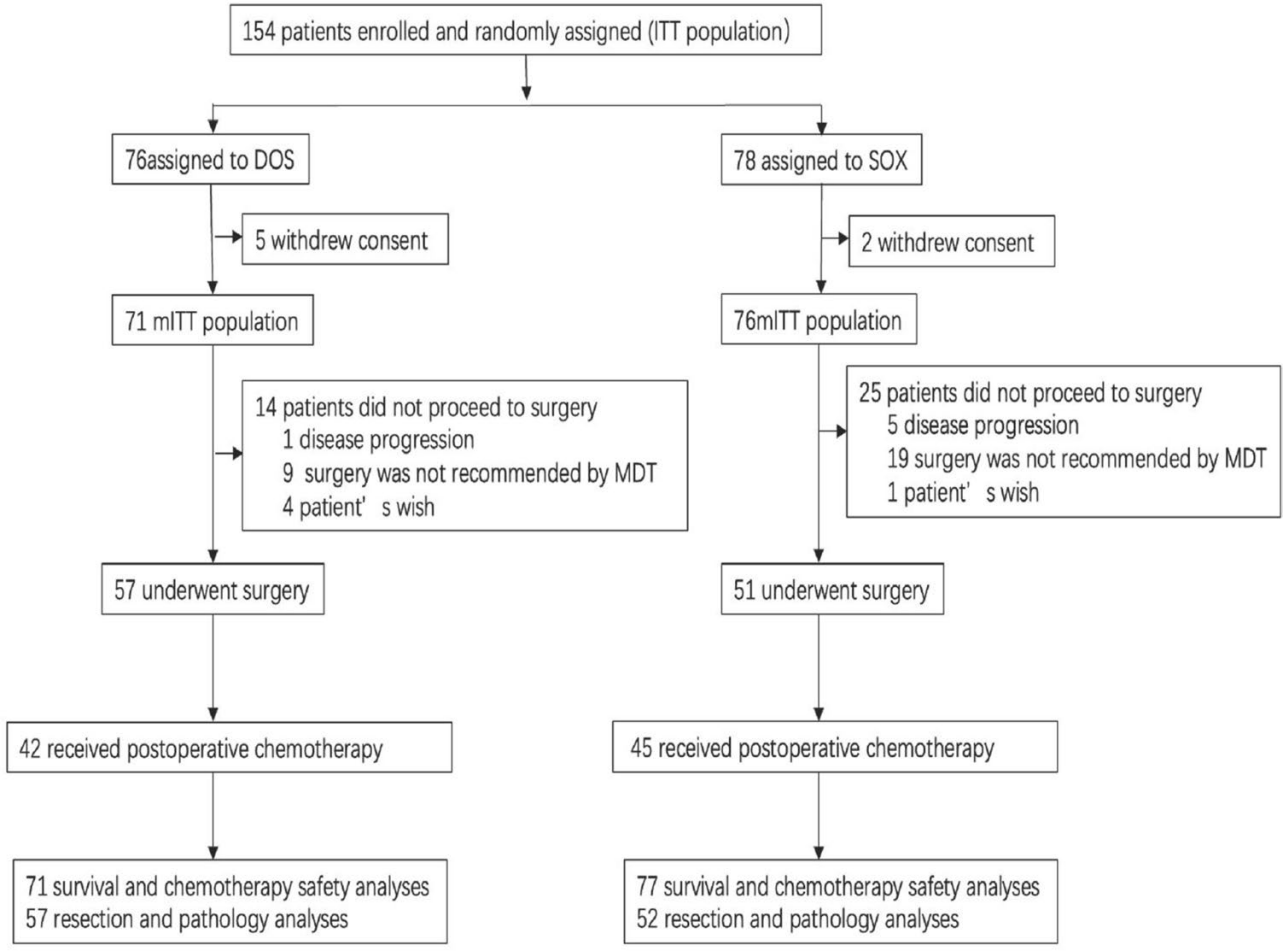
Schematic of the study profile

From Aug 2015 to Dec 2019, a total of 154 patients were initially enrolled, with 76 patients randomized to the DOS group and 78 to the SOX group. However, seven patients (DOS 5 patients; SOX 2 patients) withdrew their consent before preoperative treatment. Consequently, the mITT population consisted of 147 patients, 71 patients in the DOS group and 76 patients in the SOX group (Fig. 1). The median age of the patients was 60 (range 26–73) years old, with 78.9% (116/147) males and 21.1% (31/147) females. Baseline characteristics showed no significant difference between the two groups (Table 1). Among these patients, 39 individuals did not accept surgery (DOS 14 patients; SOX 25 patients). The reasons for not proceeding with surgery included disease progression (DOS 1 patient; SOX 4 patients), unresectable conditions due to insufficient tumor shrinkage after chemotherapy (DOS 4 patients; SOX 13 patients), identification of peritoneal metastasis during surgery (DOS 1 patient; SOX 4 patients), refusal of total gastrectomy (DOS 7 patients; SOX 4 patients) and abandonment of treatment (DOS 1 patient).

**Table 1:** Clinical characteristics of patients

	DOS (*n* = 71)	SOX (*n* = 76)	*P *value
	N (%)	N (%)	
Median age (range)	61 (26–71)	58 (35–73)	
Sex			0.69
Male	57 (80.3)	59 (77.6)	
Female	14 (19.7)	17 (22.4)	
ECOG			0.73
0	55 (77.5)	57 (75.0)	
1	16 (22.5)	19 (25.0)	
Location			0.91
Stomach	38 (53.5)	40 (52.6)	
GEJ	33 (46.5)	36 (47.4)	
Clinical tumor stage			0.89
T3	11 (15.5)	14 (18.4)	
T4a	50 (70.4)	52 (68.4)	
T4b	10 (14.1)	10 (13.2)	
Clinical nodal stage			0.35
N0	6 (8.5)	3 (3.9)	
N1	8 (11.3)	16 (21.1)	
N2	32 (45.1)	37 (48.7)	
N3a	13 (18.3)	10 (13.2)	
N3b	12 (16.9)	10 (13.2)	
Clinical TNM stage			0.43
IIA	1 (1.4)	1 (1.3)	
IIB	6 (8.5)	6 (7.9)	
IIIA	7 (9.9)	12 (21.1)	
IIIB	27 (38.0)	28 (36.8)	
IIIC	30 (42.3)	25 (32.9)	
Lauren’s type			0.83
Intestinal	28 (39.4)	29 (38.2)	
Diffuse	21 (29.6)	20 (26.3)	
Mixed	22 (31.0)	27 (35.5)	

### Pathological findings

In the mITT population, the DOS group demonstrated a significantly higher rate of MPR compared to the SOX group [25.4%, 18/71, (95% CI: 16.7–36.6) vs. 11.8%, 9/76, (95% CI: 6.4–21.0), *P* = 0.04] (Table 2), and met the primary endpoint of the study. The rates of pCR were 7% (5/71) (95% CI: 3.0–15.4) and 3.9% (3/76) (95% CI: 1.4–11.0) in the respective groups. Moreover, the DOS group had a significantly higher proportion of patients achieving R0 resection [78.9% (56/71) vs. 61.8% (47/76), *P* = 0.02]. Pathological results are shown in Table 2.

**Table 2:** Surgical and pathological results in the patients

	DOS (*n* = 71)	SOX (*n* = 76)	*P* value
mITT population	N (%)	N (%)	
TRG according to Becker TRG			
MPR (1a + 1b)	18 (25.4)	9 (11.8)	0.04
1a	6 (8.5)	3 (3.9)	
1b	12 (16.9)	6 (7.9)	
Non-MPR			
2	24 (33.8)	21 (27.6)	
3	14 (19.7)	19 (25.0)	
Unknown	1 (1.4)	2 (2.6)	
No surgery	14 (19.7)	25 (32.9)	
Resection grade			
R0 resection	56 (78.9)	47 (61.8)	0.02
Un R0 resction	15 (21.1)	29 (38.2)	
R1 resection	1 (1.4)	1 (1.3)	
R2 resection	0 (0.0)	3 (3.9)	
No surgery	14 (19.7)	25 (32.9)	

Surgery population	DOS (*n* = 57)	SOX (*n* = 51)	
Pathological tumor stage			0.72
ypT0	6 (10.5)	3 (5.9)	
ypT1a	3 (5.3)	1 (2.0)	
ypT1b	1 (1.8)	2 (3.9)	
ypT2	7 (12.3)	4 (7.8)	
ypT3	15 (26.3)	13 (25.5)	
ypT4a	21 (36.8)	26 (51.0)	
ypT4b	1 (1.8)	1 (2.0)	
Missing	3 (5.3)	1 (2.0)	
Pathological nodal stage			0.58
ypN0	19 (33.3)	18 (35.3)	
ypN1	12 (21.1)	6 (11.8)	
ypN2	12 (21.1)	10 (19.6)	
ypN3a	6 (10.5)	9 (17.6)	
ypN3b	5 (8.8)	7 (13.7)	
Missing	3 (5.3)	1 (2.0)	
Pathological TNM stage			0.83
0	5 (8.8)	3 (5.9)	
IA	4 (7.0)	3 (5.9)	
IB	2 (3.5)	3 (5.9)	
IIA	9 (15.8)	5 (9.8)	
IIB	10 (17.5)	7 (13.7)	
IIIA	7 (12.3)	7 (13.7)	
IIIB	8 (15.8)	8 (15.7)	
IIIC	9 (15.8)	14 (27.5)	
Unknown	3 (5.3)	1 (2.0)	

### Survival outcomes

**Fig. 2:**
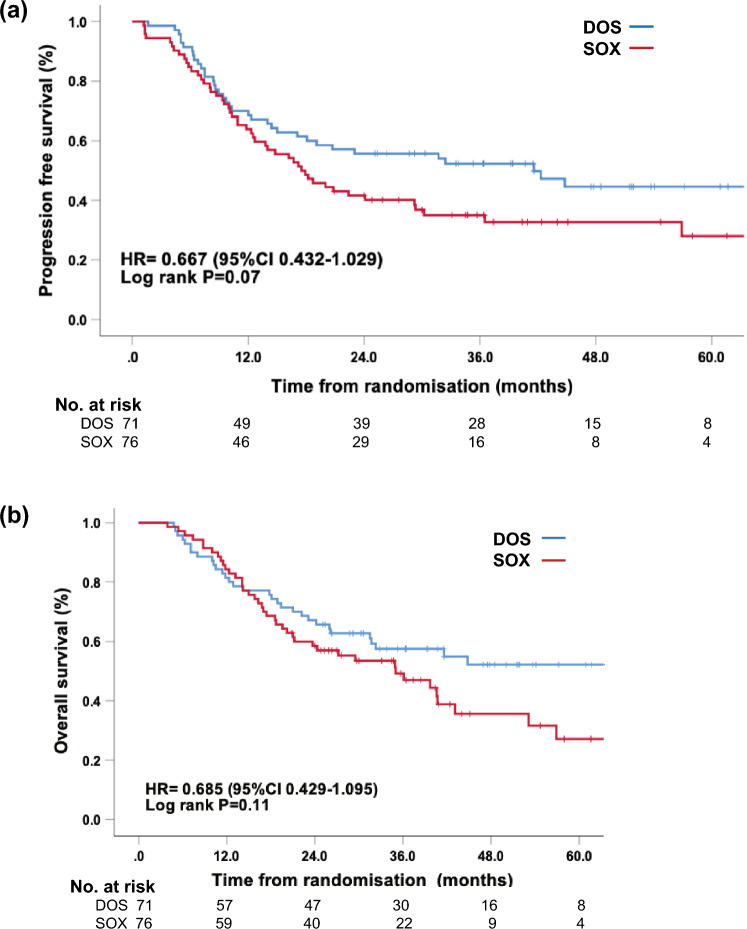
Progression free survival (**a**) and overall survival (**b**) of the mITT population

Up to Aug 24, 2021, the median follow-up time was 42.4 months (95% CI: 36.340–46.460). The 3-year PFS rates of DOS and SOX groups were 52.3 and 35.0% (HR 0.667, 95% CI: 0.432–1.029, Log-rank *P* = 0.07, Fig. 2a), respectively, while the 3-year OS rates were 57.5 and 49.2% (HR 0.685, 95% CI: 0.429–1.095, Log-rank *P* = 0.11, Fig. 2b). Patients who acquired an MPR had significantly higher 3-year PFS rate (89.4 vs. 38.6%, HR 0.076, 95% CI 0.018–0.314, Log-rank *P* < 0.001) and 3-year OS rate (100 vs. 50.1%, HR 0.024, 95% CI 0.002–0.371, Log-rank *P* < 0.001) compared to non-MPR patients (Supplementary Fig. 1 and 2). In patients who underwent surgery, distant metastases were more prevalent than local recurrence. However, it was noteworthy that there was no significant difference in the recurrence patterns between the two treatment groups. The DOS group exhibited a lower rate of local recurrence [7.0% (4/57) vs. 13.7% (7/51), *P* = 0.25] and demonstrated a tendency towards reduced distant metastases [42.1% >(24/57) vs. 58.8% (30/51), *P* = 0.08] compared with the SOX group (**Supplementary Table 1**).

### Safety profile

In the DOS group and SOX group, 85.9% (61/71) vs. 86.8% (66/76) (*P* = 0.87) and 76.1% (54/71) vs. 71.1% (54/76) (*P* = 0.49) of patients respectively completed at least three cycles and four cycles of preoperative chemotherapy, while 56.1% (32/57) and 60.8% (31/51) of the patients respectively completed at least six cycles of perioperative chemotherapy (*P* = 0.63). The most common grade 3–4 treatment-related adverse events (TRAEs) that occurred in the DOS group and SOX group were neutropenia [8.5% (6/71) vs. 10.5% (8/76), *P* = 0.67], leucopenia [1.4% (1/71) vs. 5.3% (4/76), *P* = 0.20], thrombocytopenia [1.4% (1/71) vs. 15.8% (12/76), *P* = 0.002], anemia [1.4% (1/71) vs. 3.9% (3/76), *P* = 0.34] and diarrhea [1.4% (1/71) vs. 2.6% (2/76), *P* = 0.60]. The incidence of grade 1–2 thrombocytopenia was also significantly lower in the DOS group compared to the SOX group [15.5% (11/71) vs. 31.6% (24/76), *P* = 0.02] (Table 3). During the study, no cases of febrile neutropenia or treatment-related deaths were observed. The DOS regimen didn’t pose a higher risk of surgical complications compared to the SOX regimen (**Supplementary Table 2**).

**Table 3:** Adverse events of the patients during the treatment

AEs	DOS (*n* = 71) N (%)		SOX (*n* = 76) N (%)		*P *value	
	Grade 1–2	Grade 3–4	Grade 1–2	Grade 3–4	Grade 1–2	Grade 3–4
Neutropenia	20 (28.2)	6 (8.5)	30 (39.5)	8 (10.5)	0.15	0.67
Leukopenia	27 (38.0)	1 (1.4)	34 (44.7)	4 (5.3)	0.41	0.20
Thrombocytopenia	11 (15.5)	1 (1.4)	24 (31.6)	12 (15.8)	0.02	0.002
Anemia	26 (36.6)	1 (1.4)	31 (40.3)	3 (3.9)	0.60	0.34
Nausea	29 (40.8)	0 (0.0)	38 (50.0)	1 (1.3)	0.27	0.33
Vomiting	15 (21.1)	0 (0.0)	12 (15.8)	1 (1.3)	0.40	0.33
Diarrhea	6 (8.5)	1 (1.4)	4 (5.3)	2 (2.6)	0.44	0.60
Fatigue	9 (12.7)	0 (0.0)	11 (14.5)	1 (1.3)	0.75	0.33
Serum ALT	11 (15.5)	1 (1.4)	12 (15.8)	0 (0.0)	0.96	0.30
Serum AST	11 (15.5)	0 (0.0)	11 (14.5)	0 (0.0)	0.86	NA
Hyperbilirubinemia	10 (14.1)	1 (1.4)	6 (7.9)	0 (0.0)	0.23	0.30
Peripheral neuropathy	9 (12.7)	0 (0.0)	12 (15.8)	0 (0.0)	0.30	NA

## Discussion

The patients enrolled in this study were at a relatively more advanced stage, with 30.6% classified as N3 and 37.4% as stage IIIC. These proportions were higher than those reported in previous studies, which were around 16–18 and 15%, respectively [14, 15]. The enrollment criteria for neoadjuvant gastric cancer research vary slightly between Eastern and Western studies. Western studies usually included patients with cT2, while Asian studies predominantly enrolled more advanced patients with T3–4 or those with Borrmann type 4, large type 3, or bulky N2 tumors. In this randomized, phase 2 clinical trial, preoperative DOS significantly improved the MPR rate compared to the SOX regimen (25.4 vs. 11.8%, *P* = 0.04) in patients with LAG/GEJ cancer. Furthermore, the DOS regimen showed a notable 17.1% increase in the R0 resection rate. The preoperative DOS triplet chemotherapy exhibited higher efficacy in shrinking tumors and showed a promising trend in translating this efficacy into long-term survival benefits. Compared to the SOX regimen, perioperative DOS demonstrated a 33.3% reduction in the risk of progression with numerical improvement in the 3-year PFS rate (Log-rank *P* = 0.07). Furthermore, although distant metastasis remained the main pattern of recurrence, the DOS regimen exhibited a relative advantage over SOX in reducing local recurrence and controlling distant metastasis. The local recurrence rate was as low as 7%, and the distant metastasis rate was decreased by 16.7%.

Although the DOS regimen demonstrated a higher antitumor activity compared to the SOX regimen, both groups exhibited relatively lower rates of pCR (7 vs. 3.9%) and 3-year PFS (52.3 vs. 35%). In this study, the pCR rate was defined as the proportion of patients with ypT0N0M0 in the mITT population. In the FLOT4 study, which enrolled patients with cT2, pCR rate (defined as ypT0) in mITT population was 16% with FLOT regimen [16]. While in the Asian PRODIGY and RESOLVE studies primarily included patients with cT3–4, the pCR rates (defined as ypT0N0) in surgery population for the DOS and SOX regimens were 10.4 and 5.6%, and the 3-year PFS/DFS rates were 66.3 and 59.4%, respectively [14, 15]. The recent MATTERHORN study also reported an incidence of 7% with ypT0N0 in the FLOT plus placebo arm [17]. The undesirable outcome in this study could be partly due to the enrollment of patients with more locally advanced diseases and a relatively higher proportion of patients who did not undergo surgery. Additionally, as laparoscopic exploration was not mandatory at baseline, some patients with peritoneal metastasis were included.

In addition, a significant improvement in the survival of patients who achieved MPR was observed in this study, which was consistent with previous meta-analysis findings. Gastric cancer patients with residual tumor cells <10% after neoadjuvant chemotherapy experienced better survival outcomes, with a 54% reduction in the risk of death (*P* < 0.001) [18]. Our results also provided support for the use of MPR as the surrogate primary endpoint in phase 2 studies dealing with neoadjuvant treatment in gastric cancer.

In metastatic GC, modified SOX with a reduced dose of oxaliplatin (100 mg/m^2^) had a similar efficacy compared to standard dose of CS (cisplatin plus S-1) in the first-line treatment (ORR 55.7 vs. 52.2%) [19]. Based on the finding, we designed the DOS regimen with a reduced dose of oxaliplatin (100 mg/m^2^) in this study. Actually, the completion rates of 4 cycles of preoperative chemotherapy (76.1 vs. 71.1%) and 6 or more cycles of perioperative chemotherapy (56.1 vs. 60.8%) were similar in both the DOS and SOX groups. Interestingly, the DOS regimen exhibited favorable safety without increasing toxicities compared with SOX. A lower incidence of thrombocytopenia in the DOS group was also observed in this study. The reduced dose of oxaliplatin might partly contribute to the favorable tolerance of the triplet DOS regimen. Immune-mediated reaction is one of the mechanisms of thrombocytopenia induced by oxaliplatin [20]. Steroid was reported to play a role in managing oxaliplatin-induced thrombocytopenia [21]. Therefore, premedication with dexamethasone before docetaxel might help to reduce the occurrence of thrombocytopenia to some extent. The DOS regimen did not increase the risk of postoperative complications or perioperative mortality. When compared with the FLOT4 study, the incidence of some adverse events of DOS in our study was numerically lower, mainly including grade 3–4 neutropenia and diarrhea [9]. This may be associated with the lower dose intensity of docetaxel (20 vs. 25 mg/m^2^/week) and oxaliplatin (33.3 vs. 42.5 mg/m^2^/week) [9].

Although FLOT is recommended as one of the perioperative chemotherapy regimens in China, its efficacy and safety have not been fully confirmed in Chinese population. And the inconvenience of intravenous infusion of 5-FU also contributes to the limited widespread use. However, the DOS regimen, with the oral administration of S1, is more convenient to promote. In recent years, several studies have focused on incorporating ICIs into the perioperative treatment for locally advanced GC [17, 22–25]. In the KEYNOTE-585 phase 3 trial, doublet chemotherapy plus pembrolizumab revealed a significant increase in pCR rate (12.9 vs. 2%, *P* < 0.0001) compared with chemotherapy plus placebo, while without a significant improvement in event-free survival (EFS) [26]. In the MATTERHORN phase 3 study, combining FLOT with durvalumab significantly increased the pCR rate (19 vs. 7%, *P* < 0.00001), and survival outcomes have not been reported yet [17]. In view of the favorable safety profile, it was worth further investigating DOS as a partner for ICIs in preoperative treatment.

There are several limitations in the present study. First, it was a single-center study, so the survival advantage needs to be further clarified in a multicenter phase 3 clinical trial. Second, routine diagnostic laparoscopy (DSL) was not mandatory according to the study design. Only CT scans were used to exclude peritoneal metastasis. DSL should be recommended for patients undergoing neoadjuvant treatment. Third, the fact that a relatively high proportion of patients did not receive surgery might bring bias in interpretating the data.

## Conclusions

Perioperative DOS improved MPR significantly and tended to produce better PFS compared to SOX in gastric cancer. Triplet DOS also had a favorable safety profile and could be regarded as a preferred option for perioperative chemotherapy in gastric cancer in Asia, and is worth to be further investigated in phase III study.

### Supplementary Information

Below is the link to the electronic supplementary material.

Supplementary file1 (DOCX 116 KB)

## Data Availability

Clinical data will be available from the corresponding author upon reasonable request.
