# Supplementary material for: Perioperative chemotherapy with docetaxel plus oxaliplatin and S-1 (DOS) versus oxaliplatin plus S-1 (SOX) for the treatment of locally advanced gastric or gastro-esophageal junction adenocarcinoma (MATCH): an open-label, randomized, phase 2 clinical trial
Source: Gastric Cancer. 2024 Mar 8;27(3):571–9. doi: 10.1007/s10120-024-01471-z (PMC11016518; doi:10.1007/s10120-024-01471-z)
Supplement: Supplementary file 1 — Supplementary file1 (DOCX 116 KB) [file 10120_2024_1471_MOESM1_ESM.docx]

**Supplementary Table 1. The patterns of recurrence**

|  | **DOS (*n* = 57)** | **SOX (*n* = 51)** | ***P*-value** |
| --- | --- | --- | --- |
|  | N (%) | N (%) |  |
| Local recurrence | 4 (7.0) | 7 (13.7) | 0.25 |
| Anastomotic recurrence | 1 (1.8) | 1 (2.0) | 0.94 |
| Regional lymph node metastasis | 3 (5.3) | 6 (11.8) | 0.22 |
| Distant metastasis | 24 (42.1) | 30 (58.8) | 0.08 |
| Distant lymph node metastasis | 5 (8.8) | 7 (13.7) | 0.41 |
| Organ metastasis | 8 (14.0) | 10 (19.6) | 0.44 |
| Peritoneal metastasis | 11 (19.3) | 13 (25.5) | 0.44 |

**Supplementary Table 2. Perioperative complications in patients who had surgery**

|  | **DOS (*n* = 57)** | **SOX (*n* = 51)** | | ***P-* value** | |
| --- | --- | --- | --- | --- | --- |
|  | N (%) | N (%) |  | |  |
| Death | 0 (0.0) | 0 (0.0) | NA | |  |
| Pneumonia | 1 (1.8) | 5 (9.8) | 0.07 | |  |
| Infection | 6 (10.5) | 2 (3.9) | 0.19 | |  |
| Respiratory failure | 0 (0.0) | 4 (7.8) | 0.03 | |  |
| Anastomotic leak | 1 (1.8) | 0 (0.0) | 0.34 | |  |
| Duodenal stump leak | 1 (1.8) | 0 (0.0) | 0.34 | |  |
| Mechanical ileus | 1 (1.8) | 0 (0.0) | 0.34 | |  |
| Gastrointestinal bleeding | 2 (3.5) | 1 (2.0) | 0.63 | |  |
| Phlebothrombosis | 1 (1.8) | 0 (0.0) | 0.34 | |  |

**Supplementary Fig. 1 Progression free survival depending on the pathological response**

**Supplementary Fig. 2 Overall survival depending on the pathological response**
